# Supplementary material for: Drivers of cadmium accumulation in Theobroma cacao L. beans: A quantitative synthesis of soil-plant relationships across the Cacao Belt
Source: PLoS One. 2022 Feb 2;17(2):e0261989. doi: 10.1371/journal.pone.0261989 (PMC8809552; doi:10.1371/journal.pone.0261989)
Supplement: S1 File — (DOCX) [file pone.0261989.s002.docx]

**FULL TITLE**

Drivers of cadmium accumulation in *Theobroma cacao L.* beans: a quantitative synthesis of soil-plant relationships across the Cacao Belt

**AUTHORS**

Jordon Wade^1,2*^, Marlon Ac-Pangan^3^, Vitor F. Favoretto^2^, Alexander J. Taylor^3^, Nicki Engeseth^3^, Andrew J. Margenot^2*^

^1^ Department of Crop Sciences, University of Illinois Urbana-Champaign

^2^ School of Natural Resources, University of Missouri, Columbia.

^3^ Department of Food Science and Human Nutrition, University of Illinois Urbana-Champaign

*corresponding author: [j.wade@missouri.edu](mailto:jordonwade@gmail.com); margenot@illinois.edu

**Supplementary Information**

**Search terms.**

Web of Science search was conducted on September 5th, 2020 using all available databases and the following search criteria:

*“TS=(("cadmium" OR "Cd" OR "metal*") AND ("cocoa" OR "cacao" OR "chocolate*" OR "Theobroma") AND ("soil*" OR "ground" OR "production" OR "farm*" OR "plantation*" OR "leaf" OR "leaves" OR "bean*" OR "nib*" OR "shell*")) OR AK=(("cadmium" OR "Cd" OR "metal*") AND ("cocoa" OR "cacao" OR "chocolate*" OR "Theobroma") AND ("soil*" OR "ground" OR "production" OR "farm*" OR "plantation*" OR "leaf" OR "leaves" OR "bean*" OR "nib*" OR "shell*")) OR KP=(("cadmium" OR "Cd" OR "metal*") AND ("cocoa" OR "cacao" OR "chocolate*" OR "Theobroma") AND ("soil*" OR "ground" OR "production" OR "farm*" OR "plantation*" OR "leaf" OR "leaves" OR "bean*" OR "nib*" OR "shell*")) ”*.

On October 25, 2020 both the Scopus and the CAB Abstract searches were conducted. The Scopus search used the following search criteria:

“*TITLE-ABS-KEY(("cadmium" OR "Cd" OR "metal*") AND ("cocoa" OR "cacao" OR "chocolate*" OR "Theobroma") AND ("soil*" OR "ground" OR "production" OR "farm*" OR "plantation*" OR "leaf" OR "leaves" OR "bean*" OR "nib*" OR "shell*"))* ”.

The CAB Abstracts search used the following search criteria:

“*TS=(("cadmium" OR "Cd" OR "metal*") AND ("cocoa" OR "cacao" OR "chocolate*" OR "Theobroma") AND ("soil*" OR "ground" OR "production" OR "farm*" OR "plantation*" OR "leaf" OR "leaves" OR "bean*" OR "nib*" OR "shell*")) OR TI=(("cadmium" OR "Cd" OR "metal*") AND ("cocoa" OR "cacao" OR "chocolate*" OR "Theobroma") AND ("soil*" OR "ground" OR "production" OR "farm*" OR "plantation*" OR "leaf" OR "leaves" OR "bean*" OR "nib*" OR "shell*")) OR DE=(("cadmium" OR "Cd" OR "metal*") AND ("cocoa" OR "cacao" OR "chocolate*" OR "Theobroma") AND ("soil*" OR "ground" OR "production" OR "farm*" OR "plantation*" OR "leaf" OR "leaves" OR "bean*" OR "nib*" OR "shell*")) OR BD=(("cadmium" OR "Cd" OR "metal*") AND ("cocoa" OR "cacao" OR "chocolate*" OR "Theobroma") AND ("soil*" OR "ground" OR "production" OR "farm*" OR "plantation*" OR "leaf" OR "leaves" OR "bean*" OR "nib*" OR "shell*"))* ”.

**Summary of methods to measure soil Cd.**

Across studies, there were a diversity of methods of measuring available and total soil Cd. Therefore, we aggregated the various chemical extractants to classify them as either “available” or “total” (Table S2). The “available” classification had the widest variety of methods, so we further differentiated between salt-based extractions and chelation-based extractions. Altogether, 7 studies used a salt-based extraction (e.g., NaNO_3_ or Mehlich-3) to designate available soil Cd, 9 studies used a chelation-based extraction (e.g. DTPA or EDTA), and one study used a combination of both. In contrast, all of the measures of total soil Cd included HNO_3_, (n = 19 studies), in isolation (n = 3 studies) or most commonly in conjunction with other strong acids (n = 17 studies).

**Gradient Boosted Regression Tree specifics.**

The use of boosted regression trees (BRTs) necessitates the optimization of multiple tuning parameters within the model to arrive at the final model. In choosing the potential number of splits, we used integers ranging from 1 to 9 (the total number of predictor variables in the model). Similarly, we let the minimum number of observations within each node of the individual decision trees range from 5 to 15. We specifically used stochastic gradient descent [1] and therefore also needed to optimize both the learning rate and the out-of-bag sample fraction. The learning rate, or shrinkage, ranged from 0.001 to 0.4 at intervals ranging from 0.05 to 0.02 (specific values = 0.001, 0.05, 0.075, 0.10, 0.12, 0.14, 0.16, 0.18, 0.20, 0.22, 0.24, 0.26, 0.28, 0.30, 0.325, 0.35, 0.40). The out-of-bag fraction ranged from 0.65 to 0.90 at a constant 0.05 interval. This resulted in a total of 10,098 unique permutations of our BRT model, each with 2,000 trees fit within the ensemble. We used a Gaussian distribution function. Our sample training set was a randomly selected 80% of the total dataset. Final model tuning parameters can be found in Table S3.

**Robustness check: bean Cd models.**

The presence and significant contribution (as shown by variable importance calculations) of both total soil Cd and leaf Cd introduce the possibility of a third variable problem. That is, the correlation between total soil Cd and leaf Cd (r = 0.58, *p* < 0.001; Figure S2), could be confounding our results. While BRTs largely account for this possibility, we ran separate BRT models excluding each of them to verify our findings’ robustness. The specific tuning parameters for each model can be found in Table S4. The exclusion of either total soil Cd or leaf Cd nearly doubled the model error (Table S4) and did not greatly alter the overall ranking of variable influence. Therefore, we do not believe that the correlation between total soil Cd and leaf Cd unduly influenced our results.

Partial dependence plots (PDPs) help visualize the average nonlinear effects of predictor variables within BRTs. The disadvantage of these visualizations, is that they describe the average effects of a large dataset and therefore could mask local-level heterogeneity. Individual conditional expectation (ICE) plots help to visualize the local (i.e., observation-level) robustness of global effects. They do so by graphing the average predicted outcomes for a variable of interest, while holding other variables constant [2]. We constructed centered ICE plots for each of our three predictor variables of interest—total soil Cd, soil pH, and leaf Cd—to determine our model’s robustness across the full range of values.

**Robustness check: bioconcentration factors.**

The calculation of the bioconcentration factor (BCF)—bean Cd as the numerator and total soil Cd as the denominator—introduces the potential for an inflated importance of variables that drive total soil Cd. Therefore, we ran BRT models both with and without total soil Cd as a predictor variable. The specific tuning parameters and model fits can be found in Table S4. For both models, we saw that both soil pH and soil organic carbon (SOC) were the primary driving variables, accounting for upwards of 40% and 20% of the change in model fit, respectively. The exclusion of total soil Cd from the model retained the relative rank of both leaf Cd and cation exchange capacity (CEC) as driving variables and therefore we don’t find any reason to suspect that our results are confounded by the presence or absence of total soil Cd in the model. Therefore, we have elected to include it in our finalized model to ensure that our discussion of the effects of soil pH and SOC accounts for the interrelated nature of these variables (Figure S2).

**Supporting Information References**

1. Friedman JH. Stochastic gradient boosting. Comput Stat Data Anal. 2002;38: 367–378.

2. Goldstein A, Kapelner A, Bleich J, Pitkin E. Peeking inside the black box: Visualizing statistical learning with plots of individual conditional expectation. Journal of Computational and Graphical Statistics. 2015;24: 44–65.

3. Zug KLM, Yupanqui HAH, Meyberg F, Cierjacks JS, Cierjacks A. Cadmium accumulation in Peruvian cacao (Theobroma cacao L.) and opportunities for mitigation. Water, Air, & Soil Pollution. 2019;230: 1–18.

4. Argüello D, Chavez E, Lauryssen F, Vanderschueren R, Smolders E, Montalvo D. Soil properties and agronomic factors affecting cadmium concentrations in cacao beans: A nationwide survey in Ecuador. Sci Total Environ. 2019;649: 120–127.

5. Barraza F, Maurice L, Uzu G, Becerra S, López F, Ochoa-Herrera V, et al. Distribution, contents and health risk assessment of metal(loid)s in small-scale farms in the Ecuadorian Amazon: An insight into impacts of oil activities. Science of the Total Environment. 2018;622–623: 106–120. doi:10.1016/j.scitotenv.2017.11.246

6. Ramtahal G, Yen IC, Hamid A, Bekele I, Bekele F, Maharaj K, et al. The Effect of Liming on the Availability of Cadmium in Soils and Its Uptake in Cacao (Theobroma c acao L.) In Trinidad & Tobago. Communications in Soil Science and Plant Analysis. 2018;49: 2456–2464. doi:10.1080/00103624.2018.1510955

7. Ramtahal G, Umaharan P, Hanuman A, Davis C, Ali L. The effectiveness of soil amendments, biochar and lime, in mitigating cadmium bioaccumulation in Theobroma cacao L. Sci Total Environ. 2019;693: 133563.

8. Gramlich A, Tandy S, Gauggel C, López M, Perla D, Gonzalez V, et al. Soil cadmium uptake by cocoa in Honduras. Sci Total Environ. 2018;612: 370–378.

9. Rodríguez MS, Trigozo JPR. Evaluación del contenido de metales pesados (Cd y Pb) en diferentes edades y etapas fenológicas del cultivo de cacao en dos zonas del Alto Huallaga, Huánuco (Perú). Revista de Investigación de Agroproducción Sustentable. 2017;1: 87–94. doi:10.25127/aps.20171.356

10. Huauya M, Huamani H. Edaphic macrofauna and heavy metals in the cacao crop, Theobroma cacao L. (Malvaceae). The Biologist (Lima). 2014;12: 45–55.

11. Arévalo-Gardini E, Obando-Cerpa ME, Zúñiga-Cernades LB, Arévalo-Hernández CO, Baligar V, He Z. Metales pesados en suelos de plantaciones de cacao (Theobroma cacao L.) en tres regiones del Perú. Ecología Aplicada. 2016;15: 81–89. doi:10.21704/rea.v15i2.747

12. Furcal-Beriguete P, Torres-Morales J. Vista de Determinación de concentraciones de cadmio en plantaciones de Theobroma cacao L. en Costa Rica. Revista Tecnología En Marcha. 33: 122–137. doi:10.18845/tm.v33i1.5027

13. Pedraza ET, Rojas MÁH. Distribución del contenido de cadmio en los diferentes órganos del cacao CCN-51 en suelo aluvial y residual en las localidades de Jacintillo y Ramal de Aspuzana. Revista de Investigación de Agroproducción Sustentable. 2017;1: 69–78. doi:10.25127/aps.20172.365

14. Ogunlade MO, Agbeniyi SO. Impact of pesticides use on heavy metals pollution in cocoa soils of Cross-River State, Nigeria. African Journal of Agricultural Research. 2011;6: 3725–3728. doi:10.5897/AJAR10.541

15. Lewis C, Lennon AM, Eudoxie G, Umaharan P. Genetic variation in bioaccumulation and partitioning of cadmium in Theobroma cacao L. Sci Total Environ. 2018;640–641: 696–703. doi:10.1016/j.scitotenv.2018.05.365

16. Arévalo-Gardini E, Arévalo-Hernández CO, Baligar VC, He ZL. Heavy metal accumulation in leaves and beans of cacao (Theobroma cacao L.) in major cacao growing regions in Peru. Sci Total Environ. 2017;605–606: 792–800. doi:10.1016/j.scitotenv.2017.06.122

17. Arham Z, Asmin LO, Rosmini, Nurdin M. Heavy Metal Content of Cocoa Plantation Soil in East Kolaka, Indonesia. Oriental Journal of Chemistry. 2017;33: 1164–1170.

18. Chavez E, He ZL, Stoffella PJ, Mylavarapu R, Li Y, Baligar VC. Evaluation of soil amendments as a remediation alternative for cadmium-contaminated soils under cacao plantations. Environ Sci Pollut Res Int. 2016;23: 17571–17580.

19. Bravo D, Benavides-Erazo J. The Use of a Two-Dimensional Electrical Resistivity Tomography (2D-ERT) as a Technique for Cadmium Determination in Cacao Crop Soils. Applied Sciences. 2020;10: 41–49. doi:10.3390/app10124149

20. Adewole E, Ogunmodede OT, Talabi J, Ajayi OO, Oso OA, Lajide L. Physico-chemical properties of cocoa (Theobroma cacao L. ) farm soil in Ikota, Ifedore, Nigeria. Journal of Chemical and Pharmaceutical Research. 2011;3: 544–552. Available: www.jocpr.com/articles/physicochemical-properties-of-cocoa-theobroma-cacao-l-farm-soil-in-ikota-ifedore-nigeria.pdf

21. Takrama J, Afrifa AA, Ofori-Frimpong K, Jonfia-Essien WA, Agyemang P, Galyuon I. Cadmium contamination of cocoa beans and cocoa growing agricultural soils of Ghana: There is no cause for public alarm. dokumen.tips. 2015;3: 56–61. Available: http://www.peakjournals.org/sub-journals-PJPHM.html

22. Ackah JE. Distribution of heavy metals in cocoa farm soils in the western region of Ghana. University of Ghana. 2012. Available: http://ugspace.ug.edu.gh/bitstream/handle/123456789/5812/Justice%20Edusei%20Ackah_Distribution%20of%20Heavy%20Metals%20in%20Cocoa%20Farm%20Soils%20in%20the%20Western%20Region%20of%20Ghana_2012.pdf?sequence=1&isAllowed=y

23. Agyen EK. Pesticide residue and levels of some metals in soils and cocoa beans in selected farms in the Kade area of the eastern region of Ghana. Kwame Nkrumah University of Science and Technology. 2011. Available: http://ir.knust.edu.gh/bitstream/123456789/4131/1/FINAL%20THESIS%202011%2c%20EBENEZER%20KWABENA%20AGYEN.pdf

24. Ishak CF, Osman R, Darus SZ, Rahim AA, Jusop S. Heavy metals content in soils of Peninsular Malaysia grown with cocoa and in cocoa tissues. Malaysian Journal of Soil Science. 2001;5: 47–58. Available: /paper/Heavy-metals-content-in-soils-of-Peninsular-grown-Ishak-Osman/406f7942431d1fe6978f04fd897b71c91cceeae7

25. Awokunmi EE, Ibigbami OA, Asaolu SS, Adefemi OS, Gbolagade AY. Sequential Extraction of Heavy Metals from Soil Samples Collected from Selected Cocoa Farmland in Erijiyan, Ekiti State, Nigeria. International Journal of Environmental Protection. 2015;5: 52–56. doi:10.5963/IJEP0501008

26. Bravo D, Pardo-Díaz S, Benavides-Erazo J, Rengifo-Estrada G, Braissant O, Leon-Moreno C. Cadmium and cadmium‐tolerant soil bacteria in cacao crops from northeastern Colombia. Journal of Applied Microbiology. 2018;124: 1175–1194. Available: https://sfamjournals-onlinelibrary-wiley-com.proxy2.library.illinois.edu/doi/full/10.1111/jam.13698

27. Scaccabarozzi D, Castillo L, Aromatisi A, Milne L, Búllon Castillo A, Muñoz-Rojas M. Soil, Site, and Management Factors Affecting Cadmium Concentrations in Cacao-Growing Soils. Agronomy. 2020;10: 806. doi:10.3390/agronomy10060806

28. Lajide L. Assessment of Heavy Metals Mobility in Selected Contaminated Cocoa Soils in Ondo State, Nigeria. Global Journal of Environmental Research. 2012;6: 30–35. doi:10.5829/idosi.gjer.2012.6.1.385

29. Gramlich A, Tandy S, Andres C, Chincheros Paniagua J, Armengot L, Schneider M, et al. Cadmium uptake by cocoa trees in agroforestry and monoculture systems under conventional and organic management. Sci Total Environ. 2017;580: 677–686. doi:10.1016/j.scitotenv.2016.12.014

30. Zamora ECD, Reyes-Evangelista LA, Aldoradin-Puza E, Londoñe-Bailon P, Aleman-Polo JM. Cd and Pb reduction in cocoa (Theobroma cacao) nib using two organic amendments. Revista Colombiana de Investigaciones Agroindustriales. 2020;7: 20–29. doi:10.23850/24220582.2776

31. Lewis C, Lennon AM, Eudoxie G, Sivapatham P, Umaharan P. Plant metal concentrations in Theobroma cacao as affected by soil metal availability in different soil types. Chemosphere. 2021;262: 127749. doi:10.1016/j.chemosphere.2020.127749

32. Chavez E, He ZL, Stoffella PJ, Mylavarapu RS, Li YC, Moyano B, et al. Concentration of cadmium in cacao beans and its relationship with soil cadmium in southern Ecuador. Sci Total Environ. 2015;533: 205–214. doi:10.1016/j.scitotenv.2015.06.106

33. Engbersen N, Gramlich A, Lopez M, Schwarz G, Hattendorf B, Gutierrez O, et al. Cadmium accumulation and allocation in different cacao cultivars. Sci Total Environ. 2019;678: 660–670.

| Table S2. Risk-of-bias assessment used to assess the quality of each included study. The eight criteria are reported separately, as well as an overall risk-of-bias from the study. | | | | | | | | | | |
| --- | --- | --- | --- | --- | --- | --- | --- | --- | --- | --- |
| **Study #** | **Risk-of-Bias Criteria** | | | | | | | | **Overall Risk of Bias** | **Ref.** |
|  | **Confounding** | **Selection** | **Misclassification** | **Performance** | **Detection** | **Attrition** | **Outcome**  **Reporting** | **Analysis** |  |  |
| 1 | Low | Low | Low | Low | Medium | Low | Low | High | High | [3] |
| 2 | Low | Low | Low | Low | Medium | Low | Low | Low | Medium | [4] |
| 3 | Medium | Low | Low | Low | Medium | Low | Low | High | High | [5] |
| 4 | Medium | Low | Low | Low | Medium | Low | Low | High | High | [6] |
| 5 | Low | Low | Low | Low | Medium | Low | Low | Low | Medium | [7] |
| 6 | Low | Low | Low | Low | Low | Low | Low | Low | Low | [8] |
| 7 | Low | Low | Low | Low | Medium | Low | Low | High | High | [9] |
| 8 | Medium | Low | Low | Low | Medium | Low | Low | High | High | [10] |
| 9 | Low | Low | Low | Low | Medium | Low | Low | High | High | [11] |
| 10 | Low | High | Low | Low | Low | Low | Medium | High | High | [12] |
| 11 | Low | High | Low | Low | Low | Low | Low | High | High | [13] |
| 12 | Low | High | Low | Low | Low | Low | Medium | High | High | [14] |
| 13 | Low | Low | Low | Low | Low | Low | Low | Low | Low | [15] |
| 14 | Low | Low | Low | Low | Low | Low | Low | High | Low | [16] |
| 15 | Low | Low | Low | Low | Medium | Low | Low | High | High | [17] |
| 16 | Low | Low | Low | Low | Low | Low | Low | High | High | [18] |
| 17 | Low | Low | Low | Low | Low | Low | Low | High | High | [19] |
| 18 | Low | High | Low | Low | High | Low | High | High | High | [20] |
| 19 | High | High | Low | Low | Medium | Low | High | High | High | [21] |
| 20 | Low | Low | Low | Low | Medium | Low | Medium | Medium | Medium | [22] |
| 21 | Low | High | Low | Low | Low | Low | Medium | Medium | High | [23] |
| 22 | Low | Low | Low | Low | Low | Low | Medium | High | High | [24] |
| 23 | Low | Medium | Low | Low | Low | Low | Medium | High | High | [25] |
| 24 | Low | Low | Low | Low | Low | Low | Low | Low | Low | [26] |
| 25 | Low | Medium | Low | Low | Low | Low | Low | Low | Medium | [27] |
| 26 | Low | High | Low | Low | Low | Low | Medium | High | High | [28] |
| 27 | Low | Low | Low | Low | Low | Low | Low | Low | Low | [29] |
| 28 | Low | Low | Low | Low | Low | Low | Low | Medium | Medium | [30] |
| 29 | Low | Medium | Low | Low | Low | Low | Low | Low | Medium | [31] |
| 30 | Low | Low | Low | Low | Low | Low | Low | High | High | [32] |
| 31 | Low | Low | Low | Low | Low | Low | Low | Low | Low | [33] |

| Table S3. Number of studies with each data type, by region and country. | | | | | | |
| --- | --- | --- | --- | --- | --- | --- |
| **Region** | **Country** | **# of**  **Studies** | **Soil Data (# of studies)** | | **Plant data (# of studies)** | |
|  |  |  | **Available Cd** | **Total Cd** | **Leaf Cd** | **Bean Cd** |
| West Africa | Ghana | 3 | 1 | 2 | 1 | 1 |
|  | Nigeria | 4 | 3 | 2 | 0 | 0 |
| Central and South America | Bolivia | 1 | 1 | 1 | 1 | 1 |
|  | Colombia | 2 | 0 | 2 | 0 | 0 |
|  | Costa Rica | 1 | 0 | 1 | 1 | 1 |
|  | Ecuador | 4 | 2 | 4 | 0 | 2 |
|  | Honduras | 2 | 2 | 2 | 1 | 2 |
|  | Peru | 8 | 4 | 3 | 3 | 3 |
|  | Trinidad and  Tobago | 4 | 4 | 1 | 1 | 1 |
| Southeast  Asia | Indonesia | 1 | 1 | 0 | 0 | 0 |
|  | Malaysia | 1 | 0 | 1 | 1 | 1 |

| Table S4. Extraction methods for available and total soil Cd and their prevalence in the finalized dataset. | | | |
| --- | --- | --- | --- |
| **Classification** | **Type** | **Extractant** | **# of studies**  **(# of observations)** |
| Available | Water | DI water | 1 (2) |
|  | Salt | NaNO_3_ | 1 (60) |
|  |  | Mehlich-3 extract | 4 (101) |
|  |  | AAc^a^ | 2 (26) |
|  | Chelator | EDTA^b^ | 5 (58) |
|  |  | DTPA^c^ | 4 (39) |
|  | Salt + Chelator | AAc + EDTA | 1 (15) |
| Total | Strong acid | Aqua regia only  (HNO_3_ + HCl) | 5 (657) |
|  |  | Aqua regia + other acid  (e.g. HF or H_2_O_2_) | 2 (27) |
|  |  | HNO_3_ only | 3 (28) |
|  |  | HNO_3_ + other acid  (e.g. HF or H_2_O_2_) | 7 (238) |
| ^a^  AAc = ammonium acetate (usually acidified); ^b^ EDTA = ethylenediaminetetraacetic acid; ^c^ DPTA = diethylenetriaminepentaacetic acid | | | |

| Table S5. Model tuning parameters for BRT models of both bean Cd and bioconcentration factor (BCF). Bean Cd models are the Finalized Model (presented in the main text) and models excluding either Leaf Cd (–Leaf Cd) or Total Soil Cd (–Total Soil Cd). Finalized model included both leaf Cd and total soil Cd. BCF models are either including Total Soil Cd as a predictor variable (Finalized Model presented in main text) or excluding Total Soil Cd (–Total Soil Cd). | | | | | | | |
| --- | --- | --- | --- | --- | --- | --- | --- |
| **Response**  **Variable** | **Model** | **Number**  **of trees** | **Number**  **of splits** | **Minimum observations**  **per node** | **Learning**  **rate** | **Out-of-bag**  **sampling rate** |  |
| **Bean Cd** | **Finalized model** | 11 | 9 | 12 | 0.35 | 0.65 |  |
|  | **–Leaf Cd** | 23 | 5 | 5 | 0.35 | 0.75 |  |
|  | **–Total Soil Cd** | 3 | 5 | 7 | 0.40 | 0.70 |  |
| **BCF** | **Finalized model** | 17 | 4 | 10 | 0.40 | 0.75 |  |
|  | **–Total Soil Cd** | 5 | 9 | 11 | 0.40 | 0.80 |  |

| Table S6. Relative influence of each variable and root-mean-squared error (RMSE) of BRT models of both bean Cd and bioconcentration factor (BCF). Bean Cd models are the Finalized Model (presented in the main text) and models excluding either Leaf Cd (–Leaf Cd) or Total Soil Cd (–Total Soil Cd). Finalized model included both leaf Cd and total soil Cd. BCF models are either including total soil Cd as a predictor variable (Finalized Model; presented in main text) or excluding total soil Cd (–Total Soil Cd). Values in parentheses are relative influence, which is the proportion of the reduction in error attributable to each variable. Relative importance Finalized model included both leaf Cd and total soil Cd. | | | | | |
| --- | --- | --- | --- | --- | --- |
| **Variable**  **Rank** | **Bean Cd** | | | **BCF** | |
|  | **Finalized**  **Model** | **–Leaf Cd** | **–Total Soil**  **Cd** | **Finalized**  **Model** | **–Total Soil**  **Cd** |
| 1 | Total Soil  Cd (45.8%) | Total Soil  Cd (56.7%) | Soil pH  (27.5%) | Soil pH  (44.2%) | Soil pH  (58.1%) |
| 2 | Soil pH  (26.4%) | Soil pH  (26.4%) | Leaf Cd  (25.3%) | SOC  (24.6%) | SOC  (26.3%) |
| 3 | Leaf Cd  (16.3%) | SOC  (7.5%) | SOC  (15.8%) | Total Soil  Cd (12.5%) | CEC  (10.1%) |
| 4 | SOC  (6.9%) | CEC  (4.7%) | CEC  (12.2%) | CEC  (11.8%) | Clay  (3.7%) |
| 5 | CEC  (2.0%) | Available  Soil Cd  (2.1%) | MAP  (10.3%) | Leaf Cd  (5.5%) | Leaf Cd  (1.9%) |
| 6 | Clay  (1.4%) | Clay  (1.4%) | Available  Soil Cd  (8.9%) | Clay  (1.4%) | MAP  (0.0%) |
| 7 | MAP  (0.6%) | MAP  (1.1%) | Clay  (0.0%) | MAP  (0.0%) | MAT  (0.0%) |
| 8 | Available  Soil Cd  (0.5%) | MAT  (0.1%) | MAT  (0.0%) | MAT  (0.0%) | -- |
| 9 | MAT  (0.0%) | -- | -- | -- | -- |
| Model RMSE | 0.636 | 1.147 | 1.136 | 2.237 | 2.225 |

Figure S1. Correlation matrix of predictor variables.

Figure S2. Centered individual conditional expectation (ICE) plots to visualize the local-level effects of (a) total soil Cd, (b) soil pH, and (c) leaf Cd on changes in bean Cd. Red lines indicate the global-level average marginal effects and grey lines are the local-level estimates.

Figure S3. Visualizing the effects of soil organic carbon (SOC) on bean Cd using (a) partial dependency plots and (b) centered individual conditional expectation (ICE) plots.

Figure S4. Centered individual conditional expectation (ICE) plots to visualize the local-level effects of (a) soil pH and (b) soil organic carbon (SOC) on changes in the bioconcentration factor (BCF). Red lines indicate the global-level average marginal effects and grey lines are the local-level estimates.
