# Supplementary material for: Drivers of cadmium accumulation in Theobroma cacao L. beans: A quantitative synthesis of soil-plant relationships across the Cacao Belt
Source: PLoS One. 2022 Feb 2;17(2):e0261989. doi: 10.1371/journal.pone.0261989 (PMC8809552; doi:10.1371/journal.pone.0261989)

Drivers of cacao bean Cd concentration

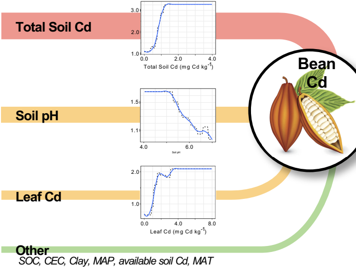

Supplement: S1 Graphical abstract — (PDF) [file pone.0261989.s003.pdf]
