# Supplementary material for: Drivers of cadmium accumulation in Theobroma cacao L. beans: A quantitative synthesis of soil-plant relationships across the Cacao Belt
Source: PLoS One. 2022 Feb 2;17(2):e0261989. doi: 10.1371/journal.pone.0261989 (PMC8809552; doi:10.1371/journal.pone.0261989)
Supplement: Attachment — Submitted filename: Comments to the Author.docx [file pone.0261989.s004.docx]

Comments to the Author

Authors have attempted a systematic review and quantitative synthesis for the detailed description of the characteristics of cacao-producing soils and edaphic and climatic drivers of bean Cd content in cacao production systems. They have identified and described multiple effects on cacao bean Cd concentration. They have also provided insight into broadly applicable mitigation strategies which can guide future research strategies.

This review manuscript is well written, however, there are some minor mistakes that must be resolved before publication.

**Abstract:**

Line 12, 13. The authors mentioned that elevated cadmium (Cd) concentrations in cacao and cocoa-based products present a potentially serious human health risk. What kind of health risks? Better to give detailed risks factors related to elevating the Cd concentration in the introduction section.

Line 19: Cacao belt referred to? The authors need to elaborate.

**Introduction**

Line 29: INTRODUCTION. (Only the first letter will be of uppercase followed by lowercase. Follow the same for other headings as well throughout the manuscript)

Line 46; replace “an” with “a”

Line 55: focus (focuses)

Line 61: remains (remain)

Line 62: at farm (at the farm)

Line 66: soil(-leaf)-bean (provide space)

Line 75: on cacao (on the cacao)

Line 76: From these insights (From these insights,) put a comma.

- Provide updated references for health risks associated with elevated concentration of Cd in Cacao and related products.
- Provide some updated references from similar studies in the introduction section

**Material and Methods:**

Line 81: on Web of Science (on the Web of Sciences)

Line 82-84: Check whether these terms will be italic or normal style?

Line 96: agreement[23,24] (create space)

Line 126: were applied (was applied)

Line 129: to estimate mean (to estimate the mean)

Line 132: GPS coordinates on a map image was performed (GPS coordinates on a map image were performed)

Line 144: characterization variable (characterization variables)

Line 146: (respectively). Remove the brackets.

Line 150: More detail is needed for Boosted regression trees (BRTs)

Line 175: RStudio[46] (create space)

**Results & Discussion**

Line 199: (Figure 3a, 3b,and 3c), (remove the comma 3b, and create space)

Line 201: 27.1ºC (create space)

Line 203: 7 ºC[55]. (create space)

Line 220: Although most of cacao’s (Although most of the cacao’s)

Line 231-233: Rewrite the sentence

Line 249: pools[2,6], (create space)

Line 277-278: rewrite the sentence and create space.

Line 292: in bean Cd were found (in bean Cd was found)

Line 362: suggest (suggests)

**Conclusion:**

Include a comprehensive and concise conclusion

**References**

- The authors need to double-check the references both in the text and bibliography
- Name of the journal should be in abbreviated form
- Check all references for journal references formate

**General comments:**

- Overall, the manuscript needs to be revised with updated references and clear methodology with proper references.
- The authors also need to check the whole manuscript for grammar, spelling and spacing mistakes to be corrected.
- The text should be in Times New Roman.
- The Pont size of the main headings (Title, Abstract, Introduction, Materials and Methods, Results and Discussion, Conclusion etc.) should be 18 according to journal formate
- **S**ubheading Pont size should be 16
- The authors need to follow the journal formate for type texting.
