# Supplementary material for: Drivers of cadmium accumulation in Theobroma cacao L. beans: A quantitative synthesis of soil-plant relationships across the Cacao Belt
Source: PLoS One. 2022 Feb 2;17(2):e0261989. doi: 10.1371/journal.pone.0261989 (PMC8809552; doi:10.1371/journal.pone.0261989)
Supplement: Attachment — Submitted filename: PONE-D-21-13459_Response to Reviewers.docx [file pone.0261989.s005.docx]

**Reviewer #1: Comments to the Author**
Authors have attempted a systematic review and quantitative synthesis for the detailed description of the characteristics of cacao-producing soils and edaphic and climatic drivers of bean Cd content in cacao production systems. They have identified and described multiple effects on cacao bean Cd concentration. They have also provided insight into broadly applicable mitigation strategies which can guide future research strategies.

This review manuscript is well written, however, there are some minor mistakes that must be resolved before publication.
We appreciate the time the reviewer has taken to read and comment on this manuscript. We will respond to each comment in red below.

Abstract:

Line 12, 13. The authors mentioned that elevated cadmium (Cd) concentrations in cacao and cocoa-based products present a potentially serious human health risk. What kind of health risks? Better to give detailed risks factors related to elevating the Cd concentration in the introduction section.

We have now added several specific examples of human health effects in the Introduction of the manuscript (L44-46).

Line 19: Cacao belt referred to? The authors need to elaborate.

We have now added the following language to better define the Cacao Belt (L22-23):

*“…we used the scientific literature to systematically compile a database of climatic, edaphic, and plant data from across the Cacao Belt, which is approximately 20 degrees latitude on either side of the equator.”*

Introduction

Line 29: INTRODUCTION. (Only the first letter will be of uppercase followed by lowercase. Follow the same for other headings as well throughout the manuscript)

We have altered the formatting here and throughout the manuscript.

Line 46; replace “an” with “a”

The suggested change has been made.

Line 55: focus (focuses)

The sentence is correct as written. Please see below for subject-verb agreement in bold.

*“To date, most* ***evaluations*** *…* ***focus*** *on specific regions”*

Line 61: remains (remain)

The suggested change has been made.

Line 62: at farm (at the farm)

The sentence is correct as written. The word “farm” is a commonly accepted scale of analysis.

Line 66: soil(-leaf)-bean (provide space)

The sentence is correct as written. The parentheses are meant to indicate the intermediate step in soil to bean translocation.

Line 75: on cacao (on the cacao)

The sentence is correct as written. Please refer to the section on count and noncount nouns here: https://owl.purdue.edu/owl/general_writing/grammar/using_articles.html.

Line 76: From these insights (From these insights,) put a comma.
The suggested change has been made.

Material and Methods:

Line 81: on Web of Science (on the Web of Sciences)

The name of the database is Web of Science, not “Web of Sciences”, maintained by Clarivate Analytics: https://www.webofknowledge.com/.

Line 82-84: Check whether these terms will be italic or normal style?

We have reformatted to all be non-italic.

Line 96: agreement[23,24] (create space)

The suggested change has been made.

Line 126: were applied (was applied)

The sentence is correct as written. Please see below for a simplified version of the subject-verb agreement (in bold).

*“…****conversions****…****were applied*** *as necessary”*

Line 129: to estimate mean (to estimate the mean)

The sentence is correct as written. Please refer to the section on count and noncount nouns here: https://owl.purdue.edu/owl/general_writing/grammar/using_articles.html.

Line 132: GPS coordinates on a map image was performed (GPS coordinates on a map image were performed)

The sentence is correct as written. Please see below for a simplified version of the subject-verb agreement (in bold).

*“Reliable* ***extraction of continuous data*** *…* ***was performed*** *using the WebPlotDigitizer.”*

Line 144: characterization variable (characterization variables)

We have amended the line to read as follows:

*“…29 studies (94%) reported at least one soil characterization variable…”*

Line 146: (respectively). Remove the brackets.

The suggested change has been made.

Line 150: More detail is needed for Boosted regression trees (BRTs)

We have added additional details to better describe how BRTs function (L181-197).

*“Boosted regression trees (BRTs) integrate the flexibility of classification and regression trees (i.e. decision trees) with the “learning” capabilities of machine learning. Decision trees iteratively search for “splits” within the data to determine thresholds of effects of independent variables on the dependent variable. However, this recursive partitioning of decision trees can overfit the model to the data. One solution to the problem of overfitting is to have a model that “learns” from previous models by both resampling and accounts for observations in previous models that were poorly described. BRTs use a “boosting” strategy to learn from previous models by fitting a succession of shallow decision trees to develop a single, combined model [40–42]. Essentially, BRTs create one “rule of thumb” from many “rules of thumb” [40]. The ability to model nonlinear relationships—specifically the ability to account for interactions between multiple nonlinear variables—and deal with missing data [43] are distinct advantages of BRTs.”*

Line 175: RStudio[46] (create space)
The suggested change has been made.

Results & Discussion
Line 199: (Figure 3a, 3b,and 3c), (remove the comma 3b, and create space)

The suggested change has been made.

Line 201: 27.1ºC (create space)

The suggested change has been made.

Line 203: 7 ºC[55]. (create space)

The suggested change has been made.

Line 220: Although most of cacao’s (Although most of the cacao’s)

The sentence is correct as written. Please refer to the section on count and noncount nouns here: https://owl.purdue.edu/owl/general_writing/grammar/using_articles.html.

Line 231-233: Rewrite the sentence

The sentence has been altered to better reflect the intended meaning.

Line 249: pools[2,6], (create space)

The suggested change has been made.

Line 277-278: rewrite the sentence and create space.

The sentence has been altered to better reflect the intended meaning.

Line 292: in bean Cd were found (in bean Cd was found)

The sentence is correct as written. Please see below for a simplified version of the subject-verb agreement (in bold).

*“… 13-fold* ***differences*** *…* ***were found****…”*

Line 362: suggest (suggests)
The suggested change has been made.

Conclusion:
Include a comprehensive and concise conclusion

We have added several sentences for a more concise and comprehensive concluding paragraph (L456-461).

References
• The authors need to double-check the references both in the text and bibliography
• Name of the journal should be in abbreviated form
• Check all references for journal references formate
We have checked and updated all references.

General comments:

• Overall, the manuscript needs to be revised with updated references and clear methodology with proper references.
• The authors also need to check the whole manuscript for grammar, spelling and spacing mistakes to be corrected.
• The text should be in Times New Roman.
• The Pont size of the main headings (Title, Abstract, Introduction, Materials and Methods, Results and Discussion, Conclusion etc.) should be 18 according to journal formate
• Subheading Pont size should be 16
• The authors need to follow the journal formate for type texting.

We have ensured that our submission conforms to journal requirements.

**Reviewer #2: Comments for authors**
The current manuscript has been structured and written well i.e. “Drivers of cadmium accumulation in Theobroma cacao L. beans: a quantitative synthesis of soil-plant relationships across the Cacao Belt”. However, I have few following concerns that authors should incorporate to improve their manuscript:

Abstract:

Line 14. Mention threshold limit for cadmium here.

The suggested change has been made.

Line 15. Provide names of few biophysical factors.

The suggested change has been made.

Results relevant to BCF should be mentioned in the abstract.

Currently, the primary driving factors of BCF (soil pH and SOC) are listed in the abstract (L38-39).

Line 26. Which soil-based strategies? Mention few ones.

The suggested change has been made.

I will recommend increasing the keywords up to 5 numbers.
The suggested change has been made.

Introduction:
Line 32. “other cocoa-containing food products are dominant exposure sources.” Which other food products? Provide please.

The suggested change has been made.

The overall formatting of the article needs to be corrected. For example, the paragraph justification in introduction section. Please check in whole manuscript and correct it.

The suggested change has been made.

Lines 39-41. “To protect human health, the EU and several countries are increasing regulations on Cd content in cocoa-containing foods.” Which other several countries? Please write names of those countries.

The suggested change has been made.

Line 46. “and offers an cash crop alternative to Erythroxylum coca.” Remove ‘an’.

The suggested change has been made.

Materials and Methods:
Methodology section is good.

Result and Discussion:
Results and discussion section is presented and discussed nicely. However, in axis of figures in some places, authors have used ‘mg/kg’ and in other places, it is ‘mg kg-1. I will recommend using ‘mg kg-1’. Please check in all figures and in whole manuscript and correct it.

We have amended the figures to have the same units in each (mg kg^-1^).

Synthesis and future work:
Lines 383-386. Please revise these lines and write in a more comprehensive way.

We have added several sentences for a more concise and comprehensive concluding paragraph (L456-461).

References:
References should be according to according to journal given format.

We have checked and updated all references.
